# Screen-based media use clusters are related to other activity behaviours and health indicators in adolescents

**DOI:** 10.1186/1471-2458-13-1174

**Published:** 2013-12-13

**Authors:** Leon Straker, Anne Smith, Beth Hands, Tim Olds, Rebecca Abbott

**Affiliations:** 1 School of Physiotherapy, Health Sciences, Curtin University, GPO Box U1987, Perth 6845, WA, Australia; 2 Institute for Health and Rehabilitation Research, The University of Notre Dame, PO Box 1225, Fremantle 6959, WA, Australia; 3 Health and Use of Time (HUT) Group, Sansom Institute for Health Research, University of South Australia, GPO Box 2471, Adelaide 5001, South Australia, Australia

**Keywords:** Sedentary behaviour, Computers, Electronic games, Physical activity, Latent class analysis

## Abstract

**Background:**

Screen-based media (SBM) occupy a considerable portion of young peoples’ discretionary leisure time. The aim of this paper was to investigate whether distinct clusters of SBM use exist, and if so, to examine the relationship of any identified clusters with other activity/sedentary behaviours and physical and mental health indicators.

**Methods:**

The data for this study come from 643 adolescents, aged 14 years, who were participating in the longitudinal Western Australian Pregnancy Cohort (Raine) Study through May 2003 to June 2006. Time spent on SBM, phone use and reading was assessed using the Multimedia Activity Recall for Children and Adults. Height, weight, muscle strength were measured at a clinic visit and the adolescents also completed questionnaires on their physical activity and psychosocial health. Latent class analysis (LCA) was used to analyse groupings of SBM use.

**Results:**

Three clusters of SBM use were found; C1 ‘instrumental computer users’ (high email use, general computer use), C2 ‘multi-modal e-gamers’ (both high console and computer game use) and C3 ‘computer e-gamers’ (high computer game use only). Television viewing was moderately high amongst all the clusters. C2 males took fewer steps than their male peers in C1 and C3 (-13,787/week, 95% CI: -4619 to -22957, p = 0.003 and -14,806, 95% CI: -5,306 to -24,305, p = 0.002) and recorded less MVPA than the C1 males (-3.5 h, 95% CI: -1.0 to -5.9, p = 0.005). There was no difference in activity levels between females in clusters C1 and C3.

**Conclusion:**

SBM use by adolescents did cluster and these clusters related differently to activity/sedentary behaviours and both physical and psychosocial health indicators. It is clear that SBM use is not a single construct and future research needs to take consideration of this if it intends to understand the impact SBM has on health.

## Background

The health benefits of being physically active in childhood and adolescence are well established [1], but it is now emerging that sedentary behaviour at this age may also impact on health. Sedentary behaviour refers to any waking behaviour characterized by an energy expenditure ≤1.5 METs while in a sitting or reclining posture [2]. Spending more than 2 hours per day in sedentary behaviours has been associated with unfavourable body composition, decreased fitness, lowered scores for self-esteem and pro-social behaviour and decreased academic achievement in school-aged children and youth [3]. Whilst being sedentary used to be regarded as the ‘opposite’ end of a physical activity continuum, they are now conceptualised as separate constructs as the behaviours are not mutually exclusive: children can be highly active and highly sedentary [4,5], or not very active but also not very sedentary [6].

Sedentary behaviour is a broad construct encompassing a range of behaviours [2]. Whilst television viewing time was widely used as a proxy measure for all sedentary behaviour in the past [7,8], over recent decades there has been a substantial increase in other sedentary activities, especially those that are screen-based such as using computers (for schoolwork, games and social networking) and playing electronic games [9]. Together these screen based media (SBM) occupy a considerable portion of young peoples’ discretionary leisure time, with recent estimates ranging from anywhere between 4 and 7 hours per day for an adolescent [10].

It is becoming apparent that the various types of SBM may be independently associated with some developmental and health risks and outcomes. For example, in a large study of Finnish adolescents [11] general computer use and playing electronic games were related to neck pain but television viewing was not. Similarly, Gopinath et al. [12] found that television viewing and playing electronic games were positively associated with diastolic blood pressure in 12-year-old children, whereas no association was found for computer use. In a national study of US children and adolescents (aged 6–17 years), television viewing was associated with poorer general health as well as poorer social/emotional health whereas reported time spent on the computer, specific use not defined, was not [13]. Babey et al. [14] also observed significantly different socio-economic and environmental correlates for television viewing compared with computer use in US adolescents. Treating SBM as a single construct may thus result in important links with health and development being missed [15]. However, even separating SBM into television viewing, computer use and electronic game use may not be sufficiently specific for understanding some relationships. Within computer use there is evidence for differential effects depending on the type of computer use. For example, computer use for gaming has been correlated with poor academic outcome [16] however computer use for communication and educational activities has been associated with better academic outcomes [17] and more favourable estimates of psychosocial stress [18].

Reducing SBM use in leisure time is regarded as a promising strategy to help prevent obesity and promote activity [19] and numerous interventions are investigating the best way to achieve this [20]. However to effectively target SBM interventions, it is essential to understand who is using what type of SBM, and also to establish whether certain SBM are associated with distinct health risks. Organising people into clusters, based on similar behaviours, may be useful to enhance the understanding of health links, identify intervention targets and tailor interventions [6,21,22].

Cluster analysis has been conducted using physical activity and sedentary behaviour exposures and has shown that children can be both physically active and engaged in substantial sedentary behaviours [4,23,24]. Recently, Ferrar et al. [25], identified over 19 studies in the past 10 years that have assessed clusters of time use patterns in adolescents. They found consistent cluster and cluster correlate patterns amongst the diverse studies, supporting the notion that this is a useful and important approach to identifying groups of people for intervention. Many of these clusters involved elements of screen time, often involving clusters which exhibited both high screen times and high levels of physical activity — the so-called “techno-actives. Indeed, Olds et al. [23] in their survey of South Australian children aged 9–14 years, found females classed as “sporties” had high SBM use and females classed as “screenies” were more active than those classed as “socialisers”, who were engaged in predominantly sedentary social activity. Nelson et al. [24], using data from the National Longitudinal Study of Adolescent Health, identified seven groups of adolescents aged 14–16 years, based on physical activity and sedentary behaviours– ranging from inactive adolescents who spent a lot of time watching television and playing games to those who did little physical activity and watched little television. Jago et al. [4] identified three clusters in British children aged 10–11 years; those who were highly active and not sedentary (more likely to be males), those who were low active and moderately sedentary (more likely to be females) and those who were both highly active and highly sedentary. Clusters, or classes, of children have also been identified using latent class analysis (LCA) [26], a model-based approach which offers several analytic advantages over standard cluster analysis [6,21]. LCA has been used successfully to identify groups of young individuals according to health risk behaviours including physical inactivity [21,27] and overall sedentary behaviour [21,22].

However, there have been no studies published to date that have used either standard cluster analysis or LCA to identify groups based solely on their SBM use, nor that have differentiated the type of computer use. Given the emerging evidence of the importance of sedentary exposure to health, a better understanding of how SBM behaviours cluster is needed. The aim of this paper therefore was to establish firstly whether distinct clusters of SBM activity exist, and secondly to examine the relationship of any identified clusters with other sedentary and physical activity behaviours and with physical and mental health indicators.

## Method

### Participants

The data for this study comes from 643 adolescents from the longitudinal Western Australian Pregnancy Cohort (Raine) Study (http://www.rainestudy.org.au). The Raine Study is an ongoing longitudinal study that started as a pregnancy cohort of women enrolled at or before the 18th week of gestation from the public antenatal clinic at the principal obstetric hospital in Perth, Western Australia, and nearby private practices. Women were enrolled from August 1989 to April 1992. A comparison of the participants completing the 17 year follow-up with Australian Bureau of Statistics census data from 2006 for families living in Western Australia with children aged 15–17 was performed to examine the representativeness of the sample with regard to sociodemographic characteristics. These were similar to the Western Australian population of families with 15- to 17-year old children, except for lower proportions of rural-dwelling families (18.4% vs. 33.9%) and of families with a combined family income of less than AUD$25,000 (7.9% vs. 10.8%), and a slightly higher proportion of urban-dwelling families living in high socioeconomic status neighbourhoods (23.6% vs. 20.6%).

The 1, 3, 5, 8, 10, 14, 17 and 20 year follow-ups have involved extensive collection of data through questionnaire and clinical examination of all children. The data for this study comes from their assessment at 14 years which took place from May 2003 to June 2006. Of the 2868 individuals originally enrolled, 1608 adolescents participated in the 14 year follow-up. Participants attended the Institute for Child Health Research for assessment and 919 agreed to attempt to complete data collection required for this study. Parents and/or guardians provided written informed consent. Curtin University Human Ethics Committee (HR84/2005) and West Australian Department of Health Ethics Committee (1172/EP) granted ethical approval for the study.

### Measures of SBM

#### Multimedia Activity Recall for Children and Adults (MARCA)

SBM use was recorded through the MARCA. The MARCA is a self-report recall electronic diary/questionnaire which requires children to recall activities in segments which can be as small as five minutes [28]. Children can choose from over 200 activities grouped into seven categories; inactivity; transport; play/sport; school work; self care; chores and ‘other’. Existing MARCA SBM categories included TV viewing and playing electronic games at a video game centre. For this study, additional activity categories were added to separate using handheld electronic game devices such as GameBoy from console devices such as PlayStation and differentiate computer use into: graphics, word processing, email, internet, gaming and general. The MARCA has been found to be a reliable self-report instrument that exhibits good content and construct validity [28]. A recent study found a correlation of rho = 0.70 and small mean differences between energy expenditure estimated by the MARCA and measured by doubly-labelled water [29]. Time spent in minutes of individual SBM activities were summed to provide a total for each day. The MARCA software was either loaded onto the participant’s computer, or if they did not have easy access to a computer they were provided with a laptop with the MARCA installed. Participants were shown how to complete the MARCA when they attended for their physical measurements (see below) and were asked to record their activities for a minimum of 7 days, then return computer, software, and data to the assessment centre. Extensive quality control of the diary data was performed including checking days with <10 or >100 activities recorded and days with <480 waking minutes. The first day’s data were excluded as it typically involved participation in the Raine study assessments. The data for participants with less than 3 weekdays and 1 weekend day data were excluded. A customised LabView program generated summary statistics for average time in each activity for each participant on weekdays, weekend days and over the whole week. In addition to SBM measures, a number of other activity and health measures were selected to examine cluster differences, based on those mentioned in the literature as relating to SBM exposure.

### Measures of other sedentary behaviours and physical activity

i) Non-SBM sedentary activity

Time spent in phone use and reading was also recorded through the MARCA with LabView processing to generate summary statistics of average weekly time spent in each activity.

ii) Physical Activity

Time spent in activities of moderate to vigorous intensity (MVPA) (defined as energy expenditure ≥ 3.0 METs [28]) were also recorded through the MARCA. Average weekly exposure was processed with the LabView program.

iii) Steps

Daily steps were measured using Yamax Digiwalker SW200 pedometers. Participants were instructed in pedometer usage when they attended for their physical measurement visit. Pedometers were worn on the right hip for at least 7 days. Daily step counts were recorded in a diary that was provided. Daily step counts below 1000 or above 40,000 were discounted. Participants with at least 4 days of acceptable data, including at least one weekend day, were included. A weekly step count was determined. Objective monitoring of physical activity using pedometers has both convergent validity [30] and construct validity [31] and the pedometer model used for the study has established reliability [32].

### Measures of physical health

i) Body mass index (BMI)

Both height (in metres) and weight (in kilograms) were measured without shoes. BMI was calculated based on the formula weight(kg)/ height(m)^2^.

ii) Back muscle endurance

Back muscle endurance was measured with a modification of the Biering-Sorenson method [33]. The Biering-Sorenson test of back muscle endurance has demonstrated moderate to high reliability correlation coefficients in both patients with low back pain and the general population [34,35]. The test has moderate construct validity, with fatigue the most common reason reported for test termination [36]. The test required participants to lie prone, with their lower body supported on a plinth, and hold their trunk level with their lower body for as long as possible [37].

iii) Sitting trunk angle

Sitting trunk angle, as a reflection of posture, was measured with the participant in a sitting position. Retro-reflective markers were fixed over the C7 and T12 spinous processes and the greater trochanter. A digital camera captured each participant’s habitual posture from a lateral perspective. Participants sat on a stool that was adjusted to popliteal height and were instructed to sit as they usually would with their gaze fixed straight ahead. Following the digitisation of each marker point using the Peak Motus motion analysis system (Peak Performance Technologies Inc, Alpharetta, GA), the angle between 2 vectors, one connecting the C7 and T12 markers and the other connecting the T12 and greater trochanter markers was calculated. This angle has good interrater reliability [38] and is representative of trunk angle in accordance with previous research [39].

iv) Neck and back pain

Participants completed a questionnaire on a laptop at the assessment centre. The questionnaire consisted of 130 questions covering a broad range of physical, medical, nutritional, psychosocial and developmental issues. The neck pain (and the same for back pain) questions were as follow: “Have you ever had neck pain?” (yes or no), “Has your neck ever been painful in the last month?” (yes or no), and “Did your neck pain last for more than 3 months” (yes or no). The full questionnaire took about one hour to complete, and the questions on neck pain and back pain occurred in the first half. Similar versions of these questions have been validated [40].

### Measures of psychosocial health

Three psychological indices were used:

i) Cowen’s Perceived Self-Efficacy Scale [41]

This questionnaire is comprised of 22 items, by which respondents rated their confidence to manage a variety of common situations on a 5-point scale ranging from 1 (not at all sure) to 5 (very sure). The questionnaire shows high internal consistency and evidence of convergent and concurrent validity [42]. Item responses were summed then averaged to provide a total score ranging from 0 to 5, with lower scores indicating less self-efficacy. Cronbach’s alpha was high for data from this study (.935).

ii) Beck’s Depression Inventory for Youth (BDI) [43]

This is a 20-item scale used to assess depressed mood in early adolescence, respondents are asked to rate the frequency of depressive symptoms from 0 to 3. The BDI for Youth has high internal consistency and excellent test-retest reliability over 7 days [43], with data from the current study also showing a high Cronbach’s alpha (.987). Responses are summed to produce a total score ranging from 0 to 60, with higher scores indicating greater depressed mood.

iii) The Youth Self-Report version of the Child Behaviour Checklist (CBCL)

This is a 112-item self-report questionnaire that measures a range of adolescent behavioural and emotional problems, and has high internal consistencies for both boys and girls [44]. Respondents rate their behaviour from 0 (not true), 1 (somewhat or sometimes true) to 2 (very or often true). Raw total scores were used for the analyses, as recommended by Achenbach [45] for research involving distinctions between children with mild symptoms. Two summary scores reflecting externalising (rule breaking and aggressive) and internalising (anxious-depressed, withdrawn, somatic) behaviours can be extracted from the questionnaire responses. The Externalising summary score contains 30 items and scores range from 0 to 60, and the Internalising summary score contains 31 items and scores range from 0 to 62, with higher scores indicating more negative behaviour. Cronbach alphas for the current study data ranged from .717 to .891 for the five syndrome scales contributing to the higher order Externalising and Internalising summary scores.

### Demographics

Socio-economic status (SES) was estimated from postcode residential address according to the Index of Advantage/ Disadvantage (IAD) from the Socio-Economic Index for Areas (SEIFA ABS). Lower scores on the IAD represent comparative disadvantage. This measure has been found to converge with individual-based measures of SES [46].

### Data analysis

#### LCA

Latent class analysis (LCA) was performed to investigate clusters of sedentary behaviour, using Latent GOLD version 4.5 (Statistical Innovations Inc, Belmont MA). To simplify model estimation due to the preponderance of zeros in the data and to reduce the influence of extreme values, five category ordinal variables of general, email, graphics, internet, word-processing and gaming computer use, non-computer game use, and television use were constructed from MARCA diary data based on thresholds shown in Table 1. Models with 1–5 classes were estimated using the eight, five category ordinal variables as class indicators and sex as an active covariate. The Latent Gold estimation involves multiple sets with random starting values to avoid local maxima. Model fit was assessed by examination and comparison of the bootstrapped p-value of the Log Likelihood (LL) statistic, Bayes Information Criterion (BIC) statistic, inspection of residual correlations within classes, and posterior probability diagnostics [47].

**Table 1:** Ordinal 5-category screen based media variables constructed from MARCA data

	**N**	**%**	**Min**	**Max**
			**(h/wk)**	**(h/wk)**
Computer-general use				
1	313	48.7	0	0
2	68	59.3	0.1	1.0
3	87	13.5	1.1	2.0
4	91	14.2	2.1	4.0
5	84	13.1	4.1	71.6
Computer-email				
1	491	76.4	0	0
2	44	6.8	0.1	1.0
3	36	5.6	1.1	3.0
4	32	5.0	3.1	7.0
5	40	6.2	7.1	27.9
Computer-graphics				
1	553	86.0	0	0
2	16	2.5	0.2	0.7
3	35	5.4	0.8	1.2
4	17	2.6	1.3	2.0
5	22	3.4	2.1	9.4
Computer-internet				
1	390	60.6	0	0
2	72	11.2	0.1	1.0
3	53	8.2	1.1	1.5
4	63	9.8	1.6	3.0
5	65	10.1	3.1	32.3
Computer-word processing				
1	532	82.7	0	0
2	36	5.6	0.1	1.0
3	21	3.3	1.1	1.5
4	17	2.6	1.6	2.0
5	37	5.8	2.1	10.9
Computer-games				
1	342	53.2	0	0
2	90	14.0	0.1	2.0
3	60	9.4	2.1	4.0
4	66	10.3	4.1	8.0
5	85	13.2	8.1	82.5
Non-computer games^				
1	412	64.1	0	0
2	57	8.9	0.1	1.0
3	53	8.2	1.1	2.5
4	65	10.1	2.6	7.0
5	56	8.7	7.2	43.0
Television				
1	66	10.3	0	6.0
2	103	16.0	6.1	10.0
3	308	47.9	10.1	23.0
4	101	15.7	23.1	32.0
5	65	10.1	32.0	56.8

Category thresholds based on first group containing zeros and remaining cases roughly equally distributed with sensible category thresholds.

^ includes console games such as PlayStation, hand held electronic games such as GameBoy and video centre game playing, although there were only 11 non-zero cases for video centre and 41 non-zero cases for hand held games

#### Cluster profile

Subjects were assigned to the latent class (cluster) for which they had the maximum posterior probability of membership. General linear models (ANOVA) or the nonparametric equivalent, Kruskal-Wallis test, were used to estimate the presence and magnitude of differences across clusters identified by LCA, in boys and girls separately, using Stata/IC 10.1 for Windows (Statacorp LP, College Station, TX). In keeping with current opinion [48],no correction of a threshold for statistical significance was made in this exploratory analysis of cluster differences. Rather p-values are presented as a guide to strength of evidence against the null hypothesis, with α = .05 considered to represent moderate evidence of cluster differences.

## Results

### Descriptive characteristics of the sample

Of the 919 adolescents who agreed to complete MARCA, 643 adolescents provided sufficient quality data for this study. The mean number of days of data per participant was 6.3 [4.5 weekdays (range 3–11); 1.8 weekend days (range 1–4)]. Those with sufficient data were more likely to be female (54% vs 46%, p < .001) and had a lower externalising subscore (10.2 vs 11.6, p < .001) than those with insufficient data but were not different in terms of SEIFA, back muscle endurance, back pain, BMI, trunk angle, depressive symptoms, self-efficacy nor TV, computer and exercise exposure. Table 2 presents characteristics of the adolescents included in the study and key descriptives of their self reported physical activity and SBM participation. Their age ranged from 13.2 to 14.5 years. Nearly all [607 (94.4%)] had at least one Caucasian parent and socioeconomic status was comparable with the general Australian population (mean SEIFA 1000). Overall median time per week on SBM was 30.1 h for boys and 21.2 h for girls.

**Table 2:** Descriptive Statistics of Study measures, by gender and overall

	**Males (n = 293)**	**Females (n = 350)**	**Overall (n = 643)**
Age (yrs: mean, SD)	14.0 (0.2)	14.0 (0.2)	14.0 (0.2)
**SBM activity** (h/wk: median, IQR)			
Computer use:			
General computer use	0 (2.2)	0.5 (2.3)	0.2 (2.2)
Email	0 (0)	0 (0.7)	0 (0)
Computer-graphics	0 (0)	0 (0)	0 (0)
Internet	0 (1.1)	0 (1.2)	0 (1.2)
Word processing	0 (0)	0 (0)	0 (0)
Computer games	1.0 (5.9)	0 (1.8)	0 (3.6)
Non-computer games	0.7 (4.5)	0 (0)	0 (1.3)
TV	17.4 (13.9)	14.3 (12.2)	15.7 (13.8)
Total SBM activity^	30.1 (19.5)	21.2 (13.3)	24.8 (16.8)
**Other activities**			
Reading (h/wk: median, IQR)	1.2 (3.0)	1.6 (3.1)	1.4 (3.1)
Phone use (h/wk: median, IQR)	0 (0.2)	0.3 (1.3)	0 (0.9)
Pedometer count (steps/wk: mean, SD)	78,854 (28,452)	68,435 (23,127)	73,149 (26,169)
Moderate/Vigorous activity (h/wk: mean, SD)	15.7 (8.4)	13.1 (6.9)	14.3 (7.7)
**Physical measures**			
Body mass index (mean, SD)	20.6 (3.7)	21.4 (4.2)	21.0 (4.0)
Back muscle endurance (secs: mean, SD)	87.0 (61.3)	90.0 (65.3)	88.5 (63.5)
Sitting trunk angle (deg: mean, SD)	238.0 (11.8)	225.6 (10.3)	231.4 (12.6)
**Spinal pain**			
Neck pain sitting (%)	14/291 (4.8%)	31/348 (8.9%)	45/639 (7.0%)
Back pain sitting (%)	31/291 (10.7%)	62/350 (17.7%)	93/641 (17.7%)
**Psychosocial measures**			
Self-efficacy^1^ (mean, SD)	3.4 (0.6)	3.3 (0.6)	3.4 (0.6)
Depression^2^ (median, IQR)	3.0 (7.0)	5.0 (8.0)	4.0 (7.0)
Internalising behaviour^3^ (mean, SD)	9.0 (6.1)	10.2 (6.5)	9.7 (6.4)
Externalising behaviour^3^ (mean, SD)	10.1 (6.1)	10.3 (6.2)	10.2 (6.2)
Socioeconomic Status based on CD^4^ (mean, SD)	1042 (91)	1036 (91)	1038 (91)

^ sum of computer, non-computer game and TV.

^1^Cowen’s Perceived Self-Efficacy Scale, possible score ranges from 1–5, higher scores indicate greater self-efficacy.

^2^Beck’s Depression Inventory, possible score ranges from 0 to 60, higher scores indicate more depressed mood.

^3^Youth Self-Report version of Child Behaviour Checklist, Internalising range 0–62, Externalising 0–60, higher scores indicate more behavioural problems.

^4^Advantage/Disadvantage (IAD) from Socio Economic Index for Areas (SEIFA) based on postcode, standardised to population mean of 1,000 with SD of 100.

### Estimation of latent classes (clusters)

**Figure 1:**
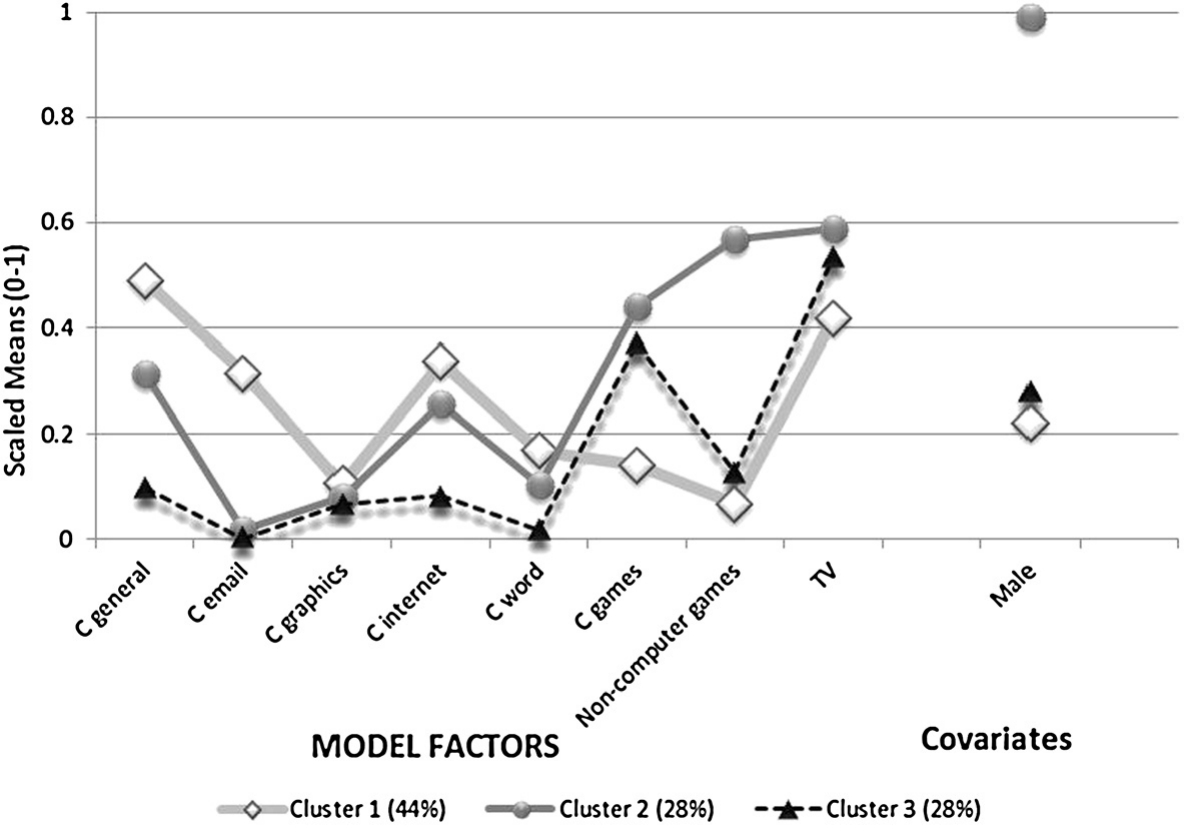
Profile plot for mean use of computer, non-computer game and television by cluster group.

Goodness-of-fit measures indicated that a three-class model represented the best fit for the data out of the one to five class models tested (Table 3). Figure 1 presents the profile plots for each cluster. The three clusters of SBM were labelled as C1 ‘instrumental computer users’ (discriminated by high email use, but also had high general computer use), C2 ‘multi-modal e-gamers’ (both high console and computer game use) and C3 ‘computer e-gamers’ (high computer game use, low general computer and internet use), and gender proportions were significantly different across the clusters (p = 0.037). C1 was estimated to be 44% of the population, C2, estimated to be 28% of the population, and C3, estimated to be 28% of the population. Participants were allocated to the cluster for which they had the maximum posterior probability of membership, which resulted in 274 (42.6%) of participants being allocated to Cluster 1 of which 58 (21.2%) were male, 185 (28.8%) to Cluster 2 of which 100% were male, and 184 (28.6%) to Cluster 3 of which 50 (27.2%) were male. Television viewing was moderately high amongst all the clusters. Table 4 presents the descriptive statistics of the sedentary behaviours used in the cluster analysis for each cluster in the units of hours per week, and the highly significant differences reflect the cluster solution derived from the ordinal transformation of the data, as expected. Computer graphics use was low and not discriminatory between groups, as reflected both in Figure 1 and Table 4.

**Table 3:** Goodness-of-fit measures for 1–5 class models

**Number of Classes**	**Number of parameters**	**Log Likelihood**	**Bootstrapped p-value of L**^**2a**^	**BIC (LL)**^**b**^	**Classification Error**^**c**^	**Entropy R-squared**^**d**^
1	32	-5,565	0.084	11,337	---	---
2	42	-5,445	0.078	11,162	0.099	0.635
3	52	-5,394	**0.204**	**11,125**	0.156	0.647
4	62	-5,381	0.138	11,163	0.190	0.629
5	72	-5,367	0.128	11,201	0.220	0.624

^a^The bootstrapped p-value of the likelihood-ratio statistic indicates if the model-based estimated frequencies are of sufficient agreement to observed frequencies, i.e. the extent to which the model fits the data, with p < 0.05 indicating a poor fit. Bootstrapping is used as the chi-squared distribution may not provide a good approximation to the distribution of the statistic, and hence provides more valid estimates. Bolded values indicate estimate of best fit.

^b^BIC adjusts the LL value for the number of parameters in the model, thus accounting for model parsimony, with a lower value indicating a more preferable model.

^c^Classification error indicates the proportion of cases that are estimated to be misclassified when cases are classified to the class for which they have the highest posterior probability, with values closer to 0 desirable.

^d^Entropy R-squared value indicates how well class membership can be predicted based on the indicator variables , with values closer to 1 desirable.

**Table 4:** Profile of identified clusters

	**C1‘Instrumental computer users’**	**C2 ‘Multi-modal E-gamers’**	**C3 ‘Computer E-gamers’**	
	**(n = 274)**	**(n = 185)**	**(n = 184)**	**p-value**^**1**^
**Gender** (n = 643)				
Male n (%)	58 (21.2)^a^	185 (100.0)^b^	50 (27.2)^a^	<0.001
Female n (%)	216 (78.8)	0	134 (72.8)	
**SBM activity** (h/week: median, interquartile range)(n = 643)				
Computer				
General comp use	1.8^a^ (3.2)	0.0^b^ (2.1)	0.0^c^ (0.0)	<0.001
Email	0.2^a^ (3.3)	0.0^b^ (0.0)	0.0^b^ (0.0)	<0.001
Computer-graphics	0.0 (0.0)	0.0 (0.0)	0.0 (0.0)	0.079
Internet	0.3^a^ (1.8)	0.0^b^ (1.4)	0.0^c^ (0.0)	<0.001
Word processing	0.0^a^ (0.5)	0.0^a^ (0.0)	0.0^b^ (0.0)	<0.001
Computer games	0.0^a^ (0.2)	2.7^b^ (7.1)	0.8^c^ (5.3)	<0.001
Non-computer games	0.0^a^ (0.0)	3.1^b^ (7.0)	0.0^a^ (0.0)	<0.001
TV	12.8^a^ (9.6)	20.9^b^ (13.8)	16.8^c^ (14.2)	<0.001
Total SBM	21.2 ^a^ (11.9)	34.1^b^ (20.4)	22.1^a^ (14.6)	<0.001
Males	24.1 ^a^ (13.1)	34.1^b^ (20.4)	22.3^a^ (17.6)	<0.001
Females	20.8 (11.7)		22.1 (14.4)	0.098
**Other activities**				
Reading (h/wk: median, IQR)(n = 643)				
Males	0.9 (2.3)	1.2 (2.9)	1.3 (3.9)	0.122
Females	1.6 (3.1)		1.7 (3.0)	0.674
Phone use (h/wk: median, IQR) (n = 643)				
Males	0.0^a^ (0.9)	0.0^b^ (0.2)	0.0^b^ (0.0)	**0.009**
Females	0.3 (1.3)		0.2 (1.2)	0.352
Pedometer count (n = 537) (steps/wk: mean, SD)				
Males	87,472^a^(27,262)	73,684^b^ (26,544)	88,490^a^ (32,191)	**<0.001**
Females	68,589 (22,455)		68,187 (24,264)	0.885
MVPA exercise out of school (n = 643)(h/wk: mean, SD)				
Males	18.2^a^ (8.4)	14.7^b^ (7.8)	16.3^a,b^ (9.7)	**0.017**
Females	12.9 (6.6)		13.5 (7.4)	0.380
**Physical measures**				
Body mass index (mean, SD)(n = 641)				
Males	20.1 (3.4)	20.7 (3.7)	20.8 (4.2)	0.536
Females	21.4 (4.0)		21.2 (4.4)	0.629
Back muscle endurance (secs: mean, SD)(n = 634)				
Males	98.9 (67.4)	81.0 (55.9)	96.0 (70.9)	0.082
Females	89.2 (63.1)		90.7 (68.9)	0.839
Sitting trunk angle (deg: mean, SD)(n = 609)				
Males	236.1 (11.6)	239.3 (11.9)	235.8 (11.1)	0.074
Females	224.9 (10.0)		226.7 (10.7)	0.126
**Spinal pain**				
Neck pain sitting (n (%))(n = 639)				
Males	3 (5.3)	10 (5.4)	1 (2.0)	0.593
Females	20 (9.4)		11 (8.2)	0.717
Back pain sitting (n (%))(n = 641)				
Males	6 (10.5)	19 (10.3)	6 (12.0)	0.943
Females	34 (15.7)		28 (20.9)	0.220
**Psychosocial measures**				
Self-efficacy^2^ (mean, SD)(n = 638)				
Males	3.5 (0.6)	3.4 (0.6)	3.4 (0.6)	0.531
Females	3.4^a^ (0.6)		3.2^b^ (0.7)	**0.028**
Depression^3^ (median, IQR)(n = 639)				
Males	3.0^a^ (4.0)	4.0^b^ (7.0)	4.0^b^ (7.0)	**0.046**
Females	5.0 (8.0)		5.0 (9.0)	0.995
Internalising behaviour^4^(mean, SD)(n = 636)				
Males	7.4 (5.0)	9.1 (6.1)	10.0 (6.8)	0.071
Females	10.2 (6.5)		10.2 (6.6)	0.950
Externalising behaviour^4^ (mean, SD)(n = 636)				
Males	9.4 (6.2)	10.3 (6.3)	10.1 (5.5)	0.647
Females	10.6(6.6)		9.8 (5.5)	0.262
**Socioeconomic Status**				
based on CD^5^ (mean, SD)(n = 625)				
Males	1073^a^ (82)	1035^b^ (91)	1029^b^ (93)	**0.016**
Females	1042 (88)		1024 (94)	0.062

^1^ANOVA used where mean (SD) reported, Kruskal-Wallis test used where median(IQR) reported, chi-squared test used where n(%) reported (or Fishers exact test where expected count <5).

^2^Cowen’s Perceived Self-Efficacy Scale, possible score ranges from 1–5, with higher scores indicating greater self-efficacy.

^3^Beck’s Depression Inventory, possible score ranges from 0 to 60, with higher scores indicating more depressed mood.

^4^Youth Self-Report version of Child Behaviour Checklist, Internalising range 0–62, Externalising 0–60, higher scores indicate more behaviouralproblems.

^5^Advantage/Disadvantage (IAD) from Socio Economic Index for Areas (SEIFA) based on postcode, standardised to population mean of 1,000 with SD of 100.

^a,b,c^ Superscripted letters define significantly different groups, ie results with different letters are significantly different.

Activity, physical and mental health variables that were significantly different between clusters have p values shown in bold.

### Latent class (cluster) profiles and demographics

For further analysis, each participant was assigned to the cluster for which they had the maximum posterior probability of membership. The mean probability of membership for the assigned cluster of participants was 0.89 for C1, 0.85 for C2 and 0.76 for C3. Within each cluster, 85%, 80% and 63% of participants assigned to C1, C2 and C3 respectively had a probability greater than or equal to 0.7 for membership of their assigned cluster. Clusters C1 and C3 were not different in total SBM exposure, even when examined separately for males and females, despite differences in general computer use, email, internet, word processing, game computer use and TV viewing (Table 4).

#### Cluster – activity/sedentary behaviour relationships

Clusters were compared across a range of behaviour and physical and psychosocial indicators (Table 4). Differences were observed amongst the males only, when comparing the clusters for the amount of MVPA reported. C2 males reported significantly fewer hours per week of MVPA than C1, with the difference between C2 and C1 estimated to be -3.5 h (95%CI: -1.0 to -5.9, p = 0.005). Similarly, C2 males took significantly fewer steps per week than either C1 (-13,787, 95%CI:-4,619 to -22,957, p = 0.003) or C3 males (-14,806, 95%CI:-5,306 to -24,305, p = 0.002). Comparisons of sedentary activities across the clusters showed differences amongst the males in phone use but not reading; C1 males reported higher phone use than their C2 or C3 peers (p = 0.009). Although the median hours of phone use was 0 in all groups, this difference can also be appreciated by the higher proportion of phone use in C1 (46.6%) to that in C2 (33.0%) and C3 (22.0%, p = 0.025). There were no differences observed in reading or phone use between C1 and C3 females.

#### Cluster – physical indicators

There were no differences in BMI between the clusters in males or females. In males, poorer back muscle endurance was observed in the C2 cluster, but the statistical evidence for this was only very weak. It was estimated that the C2 cluster achieved -18.0 secs (95%CI: -36.1 to 0.2, p = 0.053) less than the C1 cluster, and -15.0 sec (95%CI: -34.3 to 4.3, p = 0.126) less than the C3 cluster on the back muscle endurance test. Similarly, in males a greater degree of slumped sitting posture was observed in the C2 cluster, but again the statistical evidence for this was only very weak. It was estimated that the C2 cluster sat in 3.1° (95%CI: -0.5° to 6.7°, p = 0.088) more trunk flexion than the C1 cluster, and 3.5° (95%CI: -0.2° to 7.2°, p = 0.126) more trunk flexion than the C3 cluster. There was no difference for either back endurance or slumped sitting posture between C1 and C3 clusters in females. The prevalence of back and neck pain made worse by sitting did not differ between clusters, in either gender.

#### Cluster - psychosocial indicators

C1 and C3 females were similar across the majority of psychosocial indicators with the exception of self-efficacy: C1 females had marginally higher self-efficacy than C3 females (difference 0.16, 95%CI: 0.02 to 0.29, p = 0.028). Amongst the males, C1 males reported better mood, as reflected by lower depression scores (median 3.0) than either C2 (median 4.0) or C3 (median (4.0) clusters (p = 0.046). There were also differences observed in internalising behavioural problems in males between the clusters, but the statistical evidence for this was only very weak. C1 males reported less internalising behavioural problems than C2 males (difference -1.7, 95%CI: -3.5 to 0.1, p = 0.065) or C3 males (difference -2.6, 95%CI: -4.9 to -0.3, p = 0.027). There was evidence of differences in socio-economic status according to census district, with males from C1 from a higher socioeconomic district than C2 (difference 38, 95%CI: 10 to 66, p = 0.008) and C3 (difference 45, 95%CI: 9 to 80, p = 0.013).

## Discussion

This is the first study, to our knowledge, to examine whether adolescents can be grouped solely on their SBM behaviour. SBM was highly prevalent amongst these adolescents, with estimates of 27 hours/week comparable with prior findings [10]. Three defining clusters of SBM were observed: C1 ‘instrumental computer users’ who were defined by computer use for social communication, email, but also had high exposure to general computer use; C2 ‘multi-modal e-gamers’ who had both high console game use and high computer game use; and C3 ‘computer e-gamers’ who had high computer game use only. Television viewing was high in all three clusters. The findings confirm the ability to categorise adolescents by aspects of their time use, which Ferrar et al. [29] recently demonstrated across a range of time use studies from around the world. Population estimates indicated that whilst one of the clusters was exclusively male (C2 ‘multi-modal e-gamers’), two mixed gender clusters were identified, suggesting that the differences in SBM use are more than just a gender issue. Indeed although C1 and C2 had similar overall SBM exposure, they had different computer and TV use, highlighting the importance of more specific measures of computer use and of not treating SBM as a single construct. The SBM clusters identified in this study showed specific patterns of screen use behaviours that could be useful to assist in targeting of interventions aimed at reducing screen time.

The SBM clusters were shown to differ in how they correlated with other activity/ sedentary behaviours. Multi-modal e-gamer males (C2), who were distinguishable by their high time on both console games and computer games, recorded lower overall weekly steps and lower time in - MVPA than males from the other two clusters. Lower reported exercise and sport levels have also been observed in other high TV/ high video games clusters [24], though high screen time and high physical activity levels can also co-exist [4]. However screen time is often not broken down into specific activities and clustering by specific SBM activities may therefore offer some advantage. Whilst it is not known whether targeting gaming for these males would result in more activity, knowing this association could be important for helping shape interventions aimed at either activity or screen-time. Indeed it was also the C2 males that also recorded the highest TV time. Phone use was also different for the males in the ‘instrumental computer user’ cluster (C1), though perhaps this is not surprising as males who use email more may be more socially communicative in general than males who are low email users. Social clusters, demonstrating a myriad of social activities, have been identified by others [49]. Phone use was high for the females of both clusters and was therefore not able to distinguish between clusters. Females are known to spend more time on social activities more than males [50] and it was not surprising that the majority were female in the ‘instrumental computer user’ cluster, that was noted for its higher email use.

SBM clusters were also shown to relate to physical health indicators. Whilst the evidence was weak, there was a suggestion that the multi-modal e-gamers, those spending the highest time on computer and console games, were at risk of poor back muscle endurance, which may be reflective of the amount of time spent sitting in a slumped position. Computer use is widely recognised to negatively impact on neck and back posture and muscle activity [51] which could partly explain this. It was therefore surprising that there was no association between cluster grouping and neck and back pain, though previous studies looking at computer use and pain in adolescents have produced mixed findings [52,53]. It may be that it is not just the amount of computer use, but the pattern of use and extent of ergonomic mismatch between user and computer which leads to pain [54]. No associations were found between cluster groups and BMI in our study. Whilst Jago et al. [4] also found no difference in BMI between their groups which differed markedly in both physical activity and sedentary behaviours, other cluster research suggests a more consistent pattern of higher SBM activity with higher levels of overweight [25]. Overall, the activity and health profile tended to be better for those in the ‘instrumental computer user’ cluster, suggesting intervention priority should be given to targeting of the ‘multi-modal e-gamers’ and computer e-gamers’.

Psychosocial health indicators were also correlated with SBM cluster type. The ‘instrumental computer users’ females had marginally better self-efficacy than the females who spent more time on computer games. Strong relationships with peers are known to be important for adolescent self-efficacy [55]. Social networking, characterised by email use in this study (but more likely to be use of social media sites such as facebook, twitter etc. in future studies) might be reflecting this connection and management of social networks [56]. Further, the ‘instrumental computer user’ males who were less involved in computer games and more involved in emailing and phone use reported better mood, which again could be a reflection of a better social peer network. These data suggest that involving these adolescents’ social networks may be an important avenue for future SBM focussed intervention work, as has been highlighted by others in physical activity research [57]. As expected perhaps, C1 males came from higher socio-economic areas which is consistent with the research showing higher levels of child and adolescent computer game use amongst families from lower incomes [58]. Socioeconomic status appears to be related to a number of aspects of SBM related to what technology is used and for what purposes [58]. Again, the different cluster characteristics suggest clusters may help inform better-targeted interventions, in this case using social networks to target C1 adolescents and targeting computer game use in lower SES families.

### Strengths and limitations

A strength of this study is its relatively large and representative sample size. Being part of a comprehensive established longitudinal study, we were also able to explore multiple outcomes in relation to SBM use. Furthermore the MARCA, used to assess SBM, enabled a detailed characterisation of the SBM activities that adolescents were engaging in at the time of the study. This included differentiating the types of computer use. Whilst the MARCA is reliant on self-report, it has been shown to be a valid and reliable tool, and in this type of large field study, there are few alternatives for measuring the type of SBM used by adolescents.

However, being cross-sectional, the causal relationship between cluster membership and characteristics is unknown. Further, SBM and their use are evolving rapidly, both in terms of the hardware (touch screen tables and smart phones in particular) and in terms of applications (social networking and video streaming in particular). Thus the clusters identified in the current study are likely to have evolved also. What is clear from the current study is that the different types of SBM may be related differently to important health outcomes and therefore need to be considered in longitudinal studies attempting to understand the causal relationships between SBM and health and also in interventions to help make SBM more health promoting.

## Conclusion

SBM use by adolescents did cluster and these clusters related differently to other activity/sedentary behaviours and physical and psychosocial health indicators. It is clear that SBM use is not a single construct and future research needs to take consideration of the types of computer use and electronic game use in addition to TV viewing if it intends to understand the impact SBM has on health. Cluster analysis offers an attractive method to simplify this complexity and allows exploration of relationships with markers of health. Cluster analysis also offers an attractive approach to assist with the targeting interventions aiming to decrease SBM use or increase MVPA.

## Competing interests

The authors declare that they have no competing interests.

## Authors’ contributions

All authors had full access to the data and can take responsibility for the integrity of the data and accuracy of the data analyses. LS conceived the paper; AS completed full analysis of the data; LS and RA drafted the original manuscript; AS, TO and BH critiqued and added to draft manuscripts, all authors edited, critically revised and approved the final manuscript.

## Pre-publication history

The pre-publication history for this paper can be accessed here:

http://www.biomedcentral.com/1471-2458/13/1174/prepub
